# Longing for continuity: A systematic review and thematic synthesis of qualitative research on the experience of older people living with chronic illness towards the end of life

**DOI:** 10.1016/j.socscimed.2026.119220

**Published:** 2026-07

**Authors:** Emma Gobiet, Khyati Tripathi, Aline De Vleminck, Lieve Van den Block, Lara Pivodic

**Affiliations:** Vrije Universiteit Brussel (VUB) & Ghent University, End-of-Life Care Research Group, 103 Laarbeeklaan, Jette, Brussels, 1090, Belgium

**Keywords:** Chronic illness, End of life, Old age, Palliative care, Qualitative research, Systematic review, Thematic synthesis

## Abstract

Many older people spend years living with chronic illness before death. However, we lack a comprehensive understanding of how they experience and make sense of this phase of life, as knowledge about this is fragmented across diagnoses, settings, and aspects of the illness experience. This systematic review thematically synthesises qualitative research on self-reported experiences of older people living with chronic illness towards the end of life. We included 31 articles based on primary qualitative research, or mixed-methods research with separate reporting of qualitative data, describing the illness experiences of 464 older people (aged ≥65 or mean age ≥70) living with any chronic illness and nearing the end of life. We developed nine themes spread over personal, relational, and behavioural dimensions of the experience of chronic illness. Older people long to preserve a continuous sense of self, seeking ways to connect their present reality with their past sense of self and the future they envisage. Three themes closely relate to this central notion of longing for continuity: *longing for a continuous sense of self*, *needing familiarity in care*, and *striving for normalcy in daily activities*. Six other themes capture experiences more distantly related to continuity: *navigating losses*, *changing views of the future*, *feeling isolated*, *longing for independence while relying on others*, *preserving hope*, and *minimising the impact of illness*. We conclude that sustaining continuity of self is central to older people's experience of illness at the end of life, emphasising the importance of a comprehensive understanding of their realities and needs.

## Highlights

• Knowledge on older people's experience of chronic illness at the end of life is fragmented.
• We synthesise qualitative research across diagnoses, settings and areas of research.
• Older people seek continuity of self across past, present, and envisaged futures.
• This pursuit has implications for relationships, care practice, policy and society.
• Context-aware care could help sustain older people's sense of identity and meaning.

## 1 Background

Chronic illnesses or noncommunicable diseases are leading causes of death worldwide and affect people's lives over extended periods, ranging from months to decades (Mathers and Loncar, 2006). Most deaths are related to cardiovascular diseases, cancers, chronic respiratory diseases, and diabetes (World Health Organization, 2023). These chronic illnesses are especially prevalent among older people (World Health Organization, 2024). Moreover, old age is a risk factor for multimorbidity, mental health challenges, poverty, social isolation, and inadequate social support (Mohile et al., 2018; World Health Organization, 2011), all of which can complicate the impacts of chronic illness throughout its trajectory and until death. Older people may also face challenges in accessing appropriate care, and are particularly vulnerable to both over- and undertreatment (Van Den Noortgate & Van den Block, 2022). Research has shown that their preferences for end-of-life care may differ from those of younger people. They are for instance more likely to reject cardiopulmonary resuscitation (CPR) (Vilpert et al., 2023). Concurrently, the broader context in which chronic illness is experienced changes with age, as older people are often no longer professionally active, may take on caregiving roles for spouses, grandchildren or others, and frequently experience shifts in living situations, social networks, and daily routines (Kaplan, 2025; Vos-den Ouden et al., 2021). These changes make it essential to examine the experience of chronic illness towards the end of life specifically among older people, in order to understand their specific needs and ensure that they receive appropriate care and support.

We know that many older people spend years living with chronic illness before death and that the way they experience this period varies between people and changes over time. This results in a variety of changing needs and preferences (McGilton et al., 2018), and knowledge of these remains fragmented across diagnoses, settings, and areas of research. Particularly when reaching the advanced stages of chronic illness – though this is not specific to older people – a heightened awareness of one's impending death can lead to psychological distress and existential concerns (An et al., 2018). For some, such as people living with advanced cancer, the physical burden of the illness intensifies as the illness progresses, making pain management a crucial aspect of the illness experience (Yang et al., 2024). Others, for example people who have chronic kidney disease, might suffer more from the lack of control over an illness that is often perceived to be asymptomatic even in advanced stages (Nair et al., 2021). An increasing number of older people also live with multimorbidity (i.e. more than one chronic illness), creating complex and changing illness trajectories that require attention to individually specific needs (Calderón-Larrañaga et al., 2025).

However, important gaps remain in our understanding of the experience of older people who are living with chronic illness towards the end of life. First, when researching the diverse ways in which they experience living with chronic illness towards the end of life, individual studies tend to offer a valuable but partial picture by either focusing on a single diagnosis of chronic illness such as chronic obstructive pulmonary disease (Gardener et al., 2018), or one aspect of the illness experience, such as people's health and social care needs or their experiences with palliative care (McGilton et al., 2018; Sandsdalen et al., 2015). There are currently no reviews synthesising knowledge across different diagnoses, care settings, and diverse aspects of the chronic illness experience. Yet we need this knowledge to strengthen our understanding of older people's broader emotional, social, and existential experiences when nearing the end of life with chronic illness (Robinson et al., 2025). Moreover, recent research on the experience of older people who are nearing the end of life with chronic illness has highlighted that few empirical studies capture their own perspectives and that much of the existing studies have based their findings on the perspectives of professional and family caregivers (Nicholson et al., 2023; Sellars et al., 2019). While their perspectives offer important insights, it is important to gain a comprehensive understanding of the personal, subjective illness experience of older people themselves.

Qualitative research offers insights into the personal, subjective experience of chronic illness as well as contextual influences on the illness experience (Seaman et al., 2019). It has explored challenges associated with chronic illness such as loss (Lloyd et al., 2016), symptom burden (Ng et al., 2020), bodily disruption, and biographic disruption (Bury, 1982; Charmaz, 1983) in various populations. For people with cancer, for instance, the shock of diagnosis is a well-documented experience (Kirby et al., 2020), while uncertainty is a core challenge of living with multimorbidity (Etkind et al., 2022). Qualitative studies have also shed light on the diverse strategies used by people to cope with their chronic illness and associated emotions such as anxiety and loneliness (Öhman et al., 2003). This review addresses the need for a comprehensive synthesis that goes beyond specific diagnoses, care settings, and aspects of the illness experience to capture the broader, multifaceted experience of older people living with chronic illness towards the end of life in existing qualitative research.

## 2 Review aims

This systematic review aims to synthesise findings of qualitative research concerning the self-reported experience of older people living with chronic illness towards the end of life with a view to identifying overarching themes, theoretical insights, and potential research gaps (Tong et al., 2012).

## 3 Methods

### 3.1 Study design

This study is a systematic review and thematic synthesis as described by Thomas and Harden (2008). It synthesises findings from existing, primary qualitative research following the Preferred Reporting Items for Systematic Reviews and Meta-Analyses (PRISMA) statement and the Enhancing transparency in reporting the synthesis of qualitative research (ENTREQ) statement (Tong et al., 2012). The review was registered in Prospero on the 10th of June 2024 (ID: CRD42024556791).

### 3.2 Search strategy

We searched four databases – Embase, PsycINFO, PubMed and Web of Science Core Collection. Keywords were (1) older age, (2) chronic illness, (3) end of life, (4) patient perspective and (5) qualitative research. Detailed search strings for each of the four databases can be found in the supplementary materials. All authors, in consultation with an experienced librarian, finalised the keywords and search strings after thorough discussions. We conducted the search on the 4th of March 2024 and selected all publications available in the databases from inception of the database until that date for further screening. We updated the search on the 8th of September 2025 to include relevant studies on the topic published since the first search.

Two authors (EG and KT) independently screened all results of the first search by title and abstract. This was done in Rayyan, a web and mobile app for conducting systematic reviews. Differences in screening results were discussed by both authors and discussions involved a third author where necessary (LP). Both authors then screened the full texts of the selected studies to finalise the list of included studies. Discrepancies in full-text screening were discussed at length by EG and KT and, if needed, resolved with the help of the other authors. For the second search, EG screened all results by title and abstracts, while KT screened 10%. No significant discrepancies (<5%) were found in the results of their screening. EG and KT then screened all full texts of studies selected in the second search.

### 3.3 Eligibility criteria

We included primary qualitative research, or mixed-methods research in which qualitative data was separately reported, describing the illness experiences of older people (all participants aged ≥65 or a mean age of ≥70) living with any chronic illness and nearing the end of life.

We excluded:

1) systematic or scoping reviews, conference abstracts, news articles, models of care, letters, commentaries, study protocols, editorials, books, book chapters, monographs, non-peer-reviewed articles;
2) articles in languages other than English, French, Dutch or German;
3) studies focusing on the assessments of caregivers;
4) studies reporting on experiences specific to the COVID-19 pandemic;
5) studies solely describing physiological aspects of a condition, healthcare management systems or systems for disease diagnosis;
6) feasibility and acceptability studies, cost evaluation studies, intervention evaluations or evaluations of scales or assessment tools, discrete choice experiments and other studies evaluating a patient's choice for or against a treatment/procedure/intervention when they did not provide reasons for this choice.

### 3.4 Concerning the end of life

There is no universally accepted definition for the term ‘end of life’. In this review, we considered the end of life to extend beyond the terminal phase of illness, but we did not specify a time criterion. Instead, we included studies that explicitly focused on the end of life as well as studies that did not mention the end of life but included participants in advanced or severe stages of illness, and participants with inoperable or conservatively managed conditions.

We provide a detailed description and justification of the eligibility criteria, including those used to identify older people nearing the end of life, in Appendix A.

### 3.5 Quality appraisal

We used the Critical Appraisal Skills Programme Qualitative Research Checklist (CASP) to appraise the methodological quality of the selected studies (Critical Appraisal Skills Programme, 2016). The checklist includes 10 items to be answered with ‘Yes’, ‘No’ or ‘Can't tell’. The items evaluate a study's validity (e.g. Was there a clear statement of the aims of the research?), its results (e.g. Is there a clear statement of findings?) and its value (e.g. How valuable is the research?). Two authors (EG and KT) independently conducted the quality appraisal, after which they compared results.

### 3.6 Data analysis and synthesis

After full-text screening, we extracted individual characteristics from the included studies (see ‘Study characteristics’ and Table 1). We then conducted data extraction from the included studies following the three steps of thematic synthesis as described by Thomas and Harden (2008): 1) coding text, 2) developing descriptive themes, and 3) generating analytical themes.

Step 1: Coding text

**Table 1:** Characteristics of included studies.

Author(s), year, country	Study design	Population				Concept(s) studied
		Number of participants	Diagnosis of chronic illness and/or indication of the end of life	Age range	Sex or gender (w = women; m = men)	
Aasen et al. (2012), Norway	Critical discourse analysis of single interviews	11	End-stage chronic kidney disease	72-90	4w; 7m	perceptions of patient participation in their dialysis treatment of people with end-stage renal disease
Axelsson et al. (2012a), Sweden	Qualitative interpretative analysis of repeated interviews	8	Severely ill with end-stage chronic kidney disease	66-87	3w; 5m	meanings of the lived experience of being severely ill
Axelsson et al., 2012a, Axelsson et al., 2012b, Sweden	Qualitative descriptive analysis of repeated interviews					thoughts and feelings relating to death and dying
Bristowe et al. (2019), UK	Supplementary analysis of qualitative interview data	20	Stage 5 chronic kidney disease and confirmed decision for conservative management	69-95	9w; 11m	experience, impact and understanding of conservatively managed end-stage kidney disease
Selman et al. (2018), UK	Longitudinal study of cross-sectional, qualitative interviews					views and experiences of communication, information provision and treatment decision-making among patients receiving conservative care
Checa et al. (2020), Spain	Phenomenological qualitative analysis with a descriptive-interpretative approach based on single interviews	12	Stage III or IV of New York Heart Classification (NYHA) for heart failure	70-92	6w; 6m	lived experiences from a holistic perspective
Cortis and Williams (2007), UK	Qualitative inquiry for person-centered holistic perspective based on single interviews	10	Stage II, III or IV of NYHA for heart failure	80-90	5w; 5m	palliative and supportive needs; value of possible interventions
Dale and Johnston (2011), UK	Qualitative interpretive constructivist approach to interviews	6	Inoperable lung cancer in a palliative care nursing setting	67-81	4w; 2m	patients' concerns
Devik et al. (2013), Norway	Qualitative study based on narrative interviews	5	Incurable cancer, receiving life-prolonging chemotherapy	70-79	3w; 2m	meaning of the lived experience of receiving palliative treatment while living alone in a rural area
Dunham et al. (2017), UK	Interpretative phenomenological analysis of diaries and interview data	9	Diagnosis of cancer and receiving community-based specialist palliative care	67-88	2w; 7m	construction of the experience of cancer pain and how this is informed by expectations and experiences
Ek et al. (2011), Sweden	Phenomenological-hermeneutic method based on interviews, telephone conversations & field notes	4	Advanced chronic obstructive pulmonary disease and long-term oxygen therapy	66-75	3w; 1m	meaning of living with chronic obstructive pulmonary disease and long-term oxygen therapy
Franklin et al. (2006), Sweden	Hermeneutic approach based on repeated interviews	12	Nearing the end of life (no mention of specific pathologies)	85+	10w; 2m	views on dignity at the end of life in nursing homes
Frazer and Mobley (2017), US	Mixed-methods study with qualitative interviews	40	Primary diagnosis of advanced heart failure, stage 3 or 4 cancer, or advanced dementia	Mean 75.6	18w; 22m	the processes and experiences that influence quality of life ratings
Gerlich et al. (2012), Germany	Qualitative descriptive approach to single interviews	12	Heart failure in an advanced stage according to the NYHA	73-94	6w; 6m	needs and experiences with healthcare delivery
Goodman et al. (2013), UK	Exploratory qualitative study based on guided conversations	18	Dementia	68-92	13w; 5m	priorities and preferences for end-of-life care
Holmberg and Godskesen (2022), Sweden	Ethnographic and deductive study based on participant observations and interviews	19	Frailty, ≥2 diseases and assisted in daily bodily care	Mean 90.5	16w; 3m	aspects of dignity in everyday life
Hole et al. (2025), UK	Inductive thematic analysis and constant comparative techniques with semi-structured interviews	15	Chronic kidney disease with estimated glomerular filtration rate (eGFR) < 15 mL/min/1.73 m2	65-90	7w; 8m	older people's understanding and deciding between heamodialysis and conservative kidney management
House et al. (2022), US	Thematic analysis of single interviews	29	Advanced chronic kidney disease	Mean 73	10w; 19m	experiences of decision-making about treatment of advanced chronic kidney disease
Jespersen et al. (2022), Denmark	Thematic analysis of double interviews	7	Advanced gastrointestinal cancer, starting first-line palliative chemotherapy or proceeding to further treatment lines	71-81	4w; 3m	multifaceted symptoms of pain
Klindtworth et al. (2015), Germany	Qualitative longitudinal study based on repeated interviews	25	Severe heart failure according to the NYHA class III/IV	71-98	14w; 11m	perceptions of heart failure and medical, psychosocial and information needs at the end of life
Lindhardt et al. (2021), Denmark	Thematic analysis of single interviews	11	Incurable non-colorectal gastrointestinal cancer, received first-line palliative chemotherapy	65-76	6w; 5m	experiences of information on absence of curative treatment
Lindqvist et al. (2008), Sweden	Case studies based on repeated interviews	2	Hormone-refractory prostate cancer with skeletal metastases	Late 70s	2m	change of temporal awareness of past, present, and future
Lloyd et al. (2016), UK	Qualitative, longitudinal, constructivist study based on serial interviews	13	Moderately or severely frail according to the Clinical Frailty Scale (CFS)	76-92	8w; 5m	experiences, needs and priorities
(Murali et al., 2025), US	Qualitative secondary framework analysis of in-person, semi-structured interviews	17	Comorbid serious illnesses including end-stage renal disease, chronic obstructive pulmonary disease, cardiovascular disease, cancer, and cerebrovascular disease	Mean 74.2	11w; 6m	experiences and views of assisted living residents on complex care needs and serious illness communication
Russ et al. (2005), US	Phenomenological, qualitative study based on ethnographic methods	43	Chronic kidney disease and long-term renal dialysis	70-93	26w; 17m	relationships between living and quality of life, dying and the awareness of (even the desire for) death
Sugiyama and Nakamura (2025), Japan	Descriptive, qualitative research design, with semi- structured interviews to explore the lived experiences of participants	12	Diagnosis of inoperable and incurable advanced cancer	Mean 74	5w; 7m	the lives of older people living alone who are receiving outpatient cancer chemotherapy for advanced cancer
Shih et al. (2009), Taiwan	Hermeneutic inquiry based on participatory observation and interviews	35	Terminal cancer with a life expectancy of three months	Mean 75.4	16w; 19m	core constitutive patterns of experience and major foci of spiritual needs
Tavares et al. (2020), UK	Interpretative phenomenological approach to single interviews	33	Chronic obstructive pulmonary disease at different stages of the disease trajectory	Mean 72.5	11w; 22m	preferences for conversations about palliative care and future treatments with clinicians
van Gurp et al. (2020), Netherlands	Qualitative study based on single interviews	12	Incurable cancer	73-91	3w; 9m	outlooks on life and life values
Vandenberg et al. (2018), US	Secondary analysis of single interviews	15	Average of four chronic conditions, approaching the end of life with life-limiting illness	65-103	12w; 3m	phenomenon of space as experienced by assisted living residents
Warrén Stomberg (2009), Sweden	Manifest and latent content analysis of single interviews	9	Terminally ill with malignant diagnoses	w, mean 76; m, mean 71	6w; 3m	perceptions of daily life situation in hospice care

We imported the findings of all included studies into NVivo 14. EG descriptively coded the findings line-by-line for 22 of the 31 included studies (71%), while KT did so for 9 studies (29%). Both authors discussed the coding process every two weeks. Line-by-line descriptive coding of the ‘Findings’ or ‘Results’ sections of the included articles resulted in a total of 385 codes. EG and KT coded as close to the original text as possible, and formulated codes to answer the question: “How do older people experience living with chronic illness towards the end of life?” We therefore approached the illness experience as a multifaceted whole rather than aiming to exhaustively catalogue the range of older people's experiences of chronic illness.

Step 2: Developing descriptive themes

Based on recurring discussions amongst themselves and the other authors, EG and KT designed a mind map (supplementary materials), organising the descriptive codes into descriptive themes whilst illustrating connections between them.

Step 3: Generating analytical themes

Where the mind map remained a descriptive overview of all findings, the final synthesis is the result of an analytical and interpretative process involving all authors. Through regular meetings, we inferred analytical themes, attending to the relationships between interconnected descriptive themes while also considering any potentially unexpected themes (see a worked example in the supplementary materials). This interpretative step involved “abstracting out beyond the codes and themes to the larger meaning of the data” (Naeem et al., 2024, p. 11), pushing the process of analysis beyond a thematic analysis to a thematic *synthesis*.

## 4 Results

### 4.1 Literature search results

**Fig. 1:**
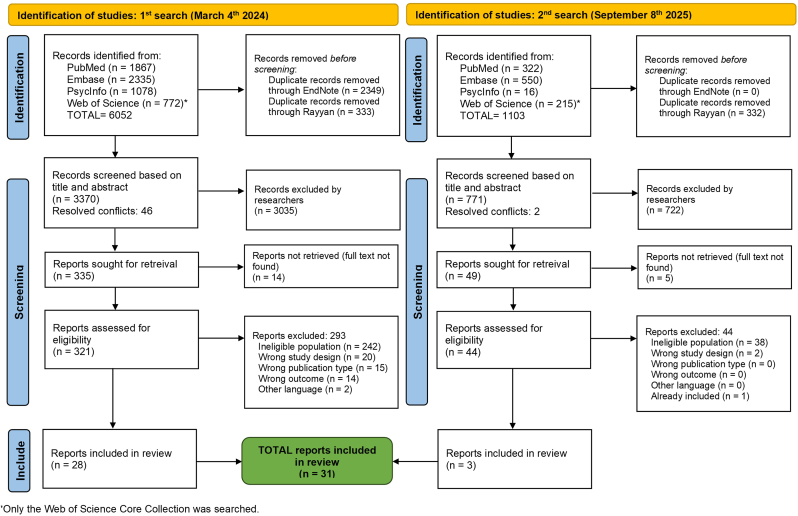
PRISMA 2020 Flow Diagram for systematic reviews based on Page et al. (2021).

Across the first and second search, we identified 7155 records through the four databases. After removing duplicates (n = 3014), we excluded 3757 records based on their titles and abstracts. Of the 384 reports left, the full texts of 19 were not found and 365 were retrieved for full-text review. Of these 365 reports, 31 met the inclusion criteria and were included in this review. Fig. 1 shows an overview of the selection process.

### 4.2 Quality appraisal

The quality appraisal using the CASP checklist showed that areas in which most studies lack clarity are those of attendance to the relationship between researchers and participants, and justification of research design and recruitment strategy (Critical Appraisal Skills Programme, 2016). On the other hand, all studies clearly justified their qualitative methodology and data collection methods. An overview of the quality appraisal can be found in the supplementary materials. We chose to retain all the selected studies in this review based on the quality appraisal.

### 4.3 Study characteristics

The studies included in this review were published between 2005 and 2025 and adopted a range of qualitative study designs. Of the 31 studies, nine were conducted in the United Kingdom (UK), seven in Sweden, five in the United States (US), two in Norway, two in Denmark, two in Germany, and one each in Spain, the Netherlands, Japan, and Taiwan. The studies included a total of 464 participants (241 women and 223 men) with various diagnoses such as chronic kidney disease, chronic heart failure, chronic obstructive pulmonary disease, frailty, dementia, and cancer (including but not limited to lung, prostate, and gastrointestinal cancer). Of the 308 participants for whom we could determine a living situation, 60 lived in nursing homes, 37 in assisted living facilities, 10 in hospices, and 201 lived at home (of whom 73 alone, 43 with their partner, 14 with other family members and 71 for whom the living situation at home was unclear). Additional study characteristics such as age, sample sizes, and concept(s) studied are summarised in Table 1.

### 4.4 Thematic synthesis of findings

#### 4.4.1 Longing for continuity

We developed nine analytical, overarching themes spread over three dimensions of the experience of chronic illness, namely the personal, relational, and behavioural dimensions. Across the findings from all studies included in this review, we found that older people living with chronic illness towards the end of life long for continuity in how they perceive themselves across past, present, and future. Striving to preserve a coherent sense of self, many people seek ways to connect their present reality with their past sense of self and the future they envisage. The longing for continuity takes on different meanings in the personal, relational, and behavioural dimensions of the chronic illness experience. As such, older people's longing for a continuous sense of self (personal dimension) influences their expectations of social relationships with professional and family caregivers (relational dimension) and the strategies they use to deal with ageing and chronic illness (behavioural dimension).

**Fig. 2:**
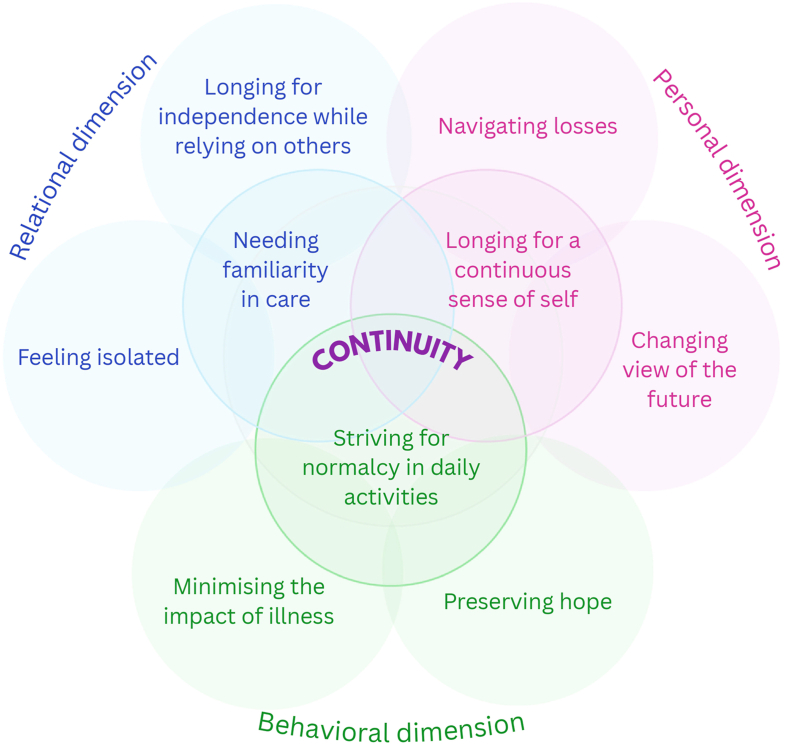
Visual representation of the nine overarching themes within the personal, relational and behavioural dimensions of the experience of chronic illness in old age.

Fig. 2 provides a visual representation of nine analytical overarching themes within the personal, relational and behavioural dimensions. Three of the themes (centre of the figure) closely relate to the key notion of a longing for continuity: *longing for a continuous sense of self*, *needing familiarity in care*, and *striving for normalcy in daily activities*. While these three central themes show conceptual overlap in relation to continuity, older people's longing for continuity takes on a different meaning in each of the three dimensions of the chronic illness experience. Six other themes surround the central themes: *navigating losses*, *changing views of the future*, *feeling isolated*, *longing for independence while relying on others*, *preserving hope*, and *minimising the impact of illness*. They fit within the personal, relational, and behavioural dimensions while being more distantly related to the longing for continuity. Codes and quotes from the original studies are provided throughout the results as illustrations. The full list of codes is archived in the VUB Institutional Data Repository (VUB/EOLC/1/000063) and can be obtained from the corresponding author upon request.

#### 4.4.2 Personal dimension

##### 4.4.2.1 Navigating losses

At the core of older people's experiences of chronic illness is the interplay between bodily decline, ageing, and sense of self. The perceived distinction between old age and illness is often blurred, with older people attributing bodily changes and ailments to either one or both. The bodily experiences they encounter – generally rooted in multiple comorbidities – result in many losses occurring either gradually or suddenly depending on the type of chronic illness and its progress.

**Code ‘Sudden deterioration experienced as frightening and overwhelming’**

With patient participants being used to a slow decay that comes with an older age, the pace of current deterioration accompanying advanced cancer frightened many participants. (van Gurp et al., 2020, p. 4)

One of the main losses is that of independence – meaning tasks and activities that used to be accomplished autonomously now require assistance from others, often giving rise to feelings of guilt, entrapment, embarrassment, vulnerability, and burdensomeness.

**Code ‘Feeling trapped by dependence’**

His loss of freedom was very distressing. *“And you see, I’m in a cage and I can’t get out.”* (Mr I, TP2) (Lloyd et al., 2016, p. 10)

**Code ‘Feeling like a burden on family’**

Being dependent also brought up feelings of being a burden. *“When I’m at my worst, then I say, it’s not worth living. ‘Don’t talk like that’, says my wife. But you do get that feeling sometimes, that you’re just a nuisance.”* (Axelsson et al., 2012a, p. 47)

Tied to the loss of independence, people also deal with the loss of, for example, plans for the future, physical mobility, privacy, and intimacy with a partner. For some, particularly older people undergoing time-consuming treatments such as haemodialysis, there is also a great sense of loss of time (i.e. time spent organising, waiting for, and getting treatment). For many, age and chronic illness also generate a distressing loss of control over one's body and over one's life.

**Code ‘Losing control over one’s life’**

This later changed when her daughter could no longer manage the care Mrs E required to lamenting her situation as alien to who she was. *“No, I think it’s a lot to do with the fact that I’m in and out of hospital and the fact that I don’t seem to have the control I had; I’m one of these people that all my life I’ve wanted to be very much in control and … right now I feel though that everything’s just getting away from me.”* (Mrs E, TP2) (Lloyd et al., 2016, p. 10)

One participant described the experience of feeling as helpless as a little baby and how that challenged that individual’s personal view of self. *“It’s horrible not being able to take care of myself. I can always get help but it’s horrible to wake up when you wet your own bed. Everything you do you’re dependent on others. For example, when you need to go to the toilet. It doesn’t feel good to ask for help going to the toilet, just like babies.”* (Franklin et al., 2006, p. 138)

**Code ‘Having no control over bodily symptoms’**

All patients experienced constant breathlessness, implying that the body was uncontrollable. Not being able to get air was an uncomfortable and painful feeling. Anna expressed her feelings about the breathlessness in this way: *“It is nasty, it is an unpleasant feeling. It is something I did not have control over.”* (Ek et al., 2011, p. 1484)

Many participants engage in a form of internal negotiation, wherein they simultaneously mourn their former abilities while accepting their present reality. This personal process of dealing with losses is not constant or uniform; rather, it evolves throughout one person's illness trajectory and differs between individuals. It is influenced by the individually-specific bodily symptoms of illness and age, social relationships and networks, and contextual resources available to them (e.g. national healthcare systems and people's financial situations).

**Code ‘Financial burden of treatment and support’**

*“The hardest part is when they don’t have a good doctor. For example, if I had money or whatever it is, after I was [unintelligible 00:46:05], if I had money for the doctor, they would give me physical therapy. I would be walking okay. You know that, right?”* (R10) (Murali et al., 2025, p. 1033)

##### 4.4.2.2 Longing for a continuous sense of self

In the face of the ongoing losses experienced due to age and illness, the perceived tension between an independent self that once was and the self that exists now generates efforts on the part of the older person to maintain a sense of continuity. To this end, they can find solace in acknowledging and celebrating past contributions and achievements, therefore reinforcing a sense of self-worth that persists despite functional decline.

**Code ‘Mourning and celebrating past life’**

It seems important to highlight the person one used to be and give credit to achievements from earlier days. One of the women says: *“I used to work a lot in a nursing home and I must have assembled at least 500 national costumes.”* Another man talks about a life of hard work and responsibility: *“I grew up on an island before the war, at the age of 14 I was treated as an adult. I have worked hard all my life, and now I benefit from that. I was in good physical shape.”* (Devik et al., 2013, p. 785)

In the present, older people want to be able to recognise themselves, partly explaining the value they attribute to doing the things they have previously always done.

**Code ‘Striving for normalcy’**

*“You often hear people talk about how valuable normal, everyday life is, don't you? I think so, too. It's something to be grateful for. Being able to spend time with my daughter. It's precious time.”* (C150) “*I have some chickens and a field. I really value them. It's good to have the things that are always important in life, rather than just disease and treatment all the time.”* (H134) (Sugiyama and Nakamura, 2025, p. 8)

The longing for a continuous sense of self also extends to the future. For example, older people fear the supposed ‘loss of self’ that possible future cognitive decline might impose on them.

**Code ‘Fearing cognitive decline’**

In particular, the fear of losing mental function, which is expected with aging and the associated loss of independence, dominates in many cases: Patient P22, T6 (89 y/o female): *“Yes, if it’s still possible. Well, my greatest wish is and always was and will be that, in old age, I hopefully will not have the experience of noticing myself that I’m losing my mind. That is one thing that would be difficult for me. Although they always say that if you are demented, you don’t notice it anyway and so on, that is a … to me, that a rather horrific thought. (…) That is already a … a burden. I think not only for us old people, but also for .. for younger people, you know. Right, but it’s better if you push it to the back of your mind and say (she laughs briefly), it will not happen to me, no.”* (Klindtworth et al., 2015, p. 5)

##### 4.4.2.3 Changing view of the future

Pain, comorbidities, and bodily changes due to age and illness serve as constant reminders of one's impending mortality, shaping expectations for the future. As physical decline progresses, older people perceive that the scope of the future shrinks. What might have seemed like a long time ahead comes much closer, transforming long-term plans and aspirations into a focus on the present and short term-goals.

**Code ‘Focusing on goals instead of losses’**

The importance of having something to aim for was identified by several of the participants. Participant 2 spoke movingly about his approaching golden wedding anniversary, which was all the more poignant as his wife suffered from dementia. *“… that’ll be 50 years married come the 22nd July, and so I set that as my target and I want to see that.”* (P2) *“It gave me something there to think about other than the worst.”* (P2) (Dale and Johnston, 2011, p. 287)

Participants also report shifting their focus from personal aspirations to broader social and existential after-death concerns, increasingly centering on what will happen after their death – how family will manage, and how one will be remembered.

**Code ‘Wishing to leave something behind for future generations’**

In his last interview, Edvin reveals that it had been his intention to write about his life, although now he is too tired to write. His strength is failing him; however, he professes indifference even though: *“I could see myself … my grandchildren, in a hundred years, great-great-grandchildren maybe … might find it, kind of interesting and … to read about what is was like … because it’s kind of a part of them … it … is, and I think it’s important to know where you’re from … so you know where you’re heading.”* (Lindqvist et al., 2008, p. 653)

Sometimes, thinking about their future is an opportunity for preparation and reflection, while at other times, it is a source of fear and uncertainty. People especially worry about leaving vulnerable loved ones behind when they are presently caring for them (e.g. a spouse living with dementia, or grandchildren with particular needs).

Most older people express the need for their remaining life to be as ‘normal’ or ‘good’ as circumstances allow, and many wish to keep living for as long as possible. Freedom from pain is an essential concern regarding the future.

**Code ‘Freedom from pain is essential’**

Patient P10, T1 (94 y/o female): *“And that [chest pain] … I don’t want to have that again.(…) No, not ever again, then the Lord God can take me back, but I couldn’t go through that again.”* (Klindtworth et al., 2015, p. 5)

Some, facing significant physical suffering caused by chronic illnesses and treatments, and feelings of loneliness or boredom, may think of or consider shortening their life.

**Code ‘Considering treatment discontinuation’**

One participant described her agony when she suffered from restrictions: *“The question is whether I draw out a few months more or less of the time that I have left. That is, in my condition, see, I don’t know how to express it, but, but for me it is almost better to die than to be suffering this way.”* (Axelsson et al., 2012b, p. 2154)

This may involve declining certain treatments or actively considering life-ending procedures. Some explicitly mention wanting life to end, while others express curiosity or even hope about death because it could reunite them with deceased loved ones. When considering the future, older people with chronic kidney disease or cancer sometimes mention a conscious weighing-up of the benefits of potentially life-prolonging treatments such as dialysis against the negative effects of these treatments on their quality of life.

**Code ‘Balancing quality of life and life expectation in treatment choice’**

The assumed extension to life provided by dialysis needed to be of acceptable quality, and participants' capability to live and undertake activities independently appeared to be critically important: *“If it's going to give me a reasonable quality of life, then it will keep me going. If I didn't think that I would have a reasonable quality of life, then I would take the option of nothing.”* (Muriel; 60s; HD) (Hole et al., 2025, p. 5)

2) Relational dimension

#### 4.4.3 Relational dimension

##### 4.4.3.1 Longing for independence while relying on others

While older people living with chronic illness may desire independence, the consequences of their illness and age create a necessary reliance on both professional and family caregivers. Thus, older people negotiate maintaining desired levels of independence and control, and placing trust in others to provide care that aligns with their needs and preferences, while striving to maintain a sense of self. In this sense, the wish for independence and control over what happens in their own lives seems to stem from the aforementioned need for continuity between one's healthier past self and present reality. Striving for independence and control seems to serve as confirmation that they are still themselves. In practice, this translates to older people often expressing the need to retain control over certain aspects of their daily lives or care processes, and taking pride in independence when they can retain it (e.g. dressing independently).

**Code ‘Taking pride in independence’**

In some cases, maintaining autonomy encourages coping through mobilizing personal resources. One man describes this ability with much pride: *“I have been alone all my life so I’m used to taking care of myself. I travel alone to treatment of course - I’m not yet that poorly.”* (Devik et al., 2013, p. 784)

While navigating the complex emotional terrain of needing help while striving to maintain a dignified sense of self, participants express gratitude for the support they receive from family, friends, and healthcare professionals. Loved ones in particular provide crucial emotional support, and while social networks often seem to diminish as illness progresses (e.g. due to restrictions in mobility, tiredness, or the unpredictability of symptoms), people report intensified or deepened relationships with the people they are closest to, though it remains difficult to discuss deterioration and death with them. Healthcare professionals sometimes act as middle-men in communicating the older people's needs to loved ones.

**Code ‘Healthcare professionals as middle man in communicating needs to family’**

Thirty participants (86%) expressed the need for affirmation from their significant others of the positive worldly contributions they had made during their lives on others and society, coupled with face-to-face verbalizations of intimate feelings with significant others. Nurses assisted in this process by communicating the importance to participants and their significant others, which was greatly appreciated. [ …] *“With the nurse’s encouragement, my children told me that they needed me so much. I know it is difficult for us Taiwanese to say so. As a dying person, I’m so content and this has reaffirmed my strong sense of belonging.”* (Shih et al., 2009, p. E35)

The presence of loved ones can also drive older people to accept to continue enduring the consequences of age, chronic illness, and treatments.

**Code ‘Family gives meaning to life-prolonging treatment’**

The inner meaning of a life with haemodialysis was related to having a caring family. Being able to see children and grandchildren progress in life for a bit longer was the evident and increasing meaning for living with, and for enduring, dialysis treatment. (Axelsson et al., 2012a, p. 49)

*“I couldn’t make a decision on therapy options without the help of my family and friends. Without them, I just wanted to die immediately.”* (Shih et al., 2009, p. E34)

Additionally, loved ones can provide valuable support when interactions with healthcare systems are complicated.

##### 4.4.3.2 Needing familiarity in care

Interactions with the healthcare system significantly impact experiences of age and chronic illness, as people living with chronic illness are in frequent contact with healthcare systems for extended periods of time. In the relational dimension of their chronic illness experience, older people's longing for continuity takes on the very concrete meaning of needing to be cared for by familiar people who can consider their past, present, and future as fully relevant to the care process.

**Code ‘Fragmented care delivery’**

*“When the place first opened, 11 people worked in the wellness center. Out of those 11, we only have 2 left, so they have 9 new people. When you talkin’ to some of ‘em, they have no idea what you’re talkin’ about, so it’s a matter of there’s no way you can literally communicate with ‘em, so you have to do certain things yourself, but that’s what it is.”* (R11) (Murali et al., 2025, p. 1033)

While older people generally trust healthcare professionals to know what they are doing and make the right decisions regarding care, trust is further reinforced when they perceive empathy, time, and continuity of care throughout their numerous interactions with healthcare systems. Conversely, older people sometimes struggle to be listened to, and can feel ignored or objectified within medical settings.

**Code ‘Feeling objectified by healthcare professionals’**

*“You become an object when you don’t get information beforehand. The doctors have an individual round in one room and the patient is an object that can neither influence nor understand anything.”* Being depersonalized was described to happen particularly during hospitalizations, but occurred also in the dialysis clinic. (Axelsson et al., 2012a, p. 49)

They relate these feelings to healthcare professionals’ lack of time, or their lack of consideration for the impact of comorbidities and non-medical concerns.

**Code ‘Healthcare professionals lack understanding of broader illness experience’**

Patients also perceived clinicians as overly focused on “the disease that they walked in the room for” and that interactions often consisted of a series of closed-ended questions posed by clinicians intended to assess whether patients fit a “textbook” presentation of chronic kidney disease. (House et al., 2022, p. 7)

Clinicians and patients were described as *“in the hands of a mystery”* (George); for example, George wanted to know what caused his terrible itching, Alice worried about getting water on her lungs, and Michael felt renal clinicians didn’t understand his other health problems or their interaction with his renal disease (*“it’s like they know one bit of you, but not the other bits, if you know what I mean”*). (Selman et al., 2018, p. 7)

A lack of continuity in care and perceived disregard for older people's personal experiences, such as fear of falling or difficult living situations, contribute to frustration and disengagement from care. Sometimes, older people feel that the care they receive is substandard because they are too old to receive ‘good’ care.

**Code ‘Feeling discriminated against in treatment options’**

This man assumed that because of his age, others would make the decision not to treat any future illnesses: *“And people of my age, they don’t [admit to hospital] … they just let us kick the bucket, don’t they? Do you know what I mean by ‘kick the bucket’?”* [James] (Goodman et al., 2013, p. 1643)

Familiarity with healthcare professionals fosters a sense of security and facilitates conversations about illness progression and end-of-life concerns because people feel more comfortable to address such topics with professional caregivers they know. Regarding information and communication (about illness diagnosis, prognosis, progression, and death), older people express varying needs and preferences. Palliative care needs are similarly variable and change over time. We chose not to describe the variability in people's specific care preferences in detail in this review as it focuses on emotional, psychological, and existential experiences which go beyond older people's information and communication needs or specific palliative care needs (see Auriemma et al., 2014; González-González et al., 2021; Robinson et al., 2025 for reviews on these topics). When it comes to conversations about the end of life and palliative care, however, most older people express the need to trust their caregivers on a personal level – such trust is best built through continuity in care and familiarity.

**Code ‘Importance of trust for communication about impending death’**

The importance of trust and continuity was described. The significance of talking to the right person and of healthcare professionals showing interest was emphasised. (Axelsson et al., 2012b, p. 2154)

Additionally, in order to share their end-of-life and palliative care needs with healthcare professionals, older people need to believe that they have the competence and time to address such issues. They are not always convinced that specific healthcare professionals have the authority or responsibility required to implement their end-of-life preferences.

**Code 'Expert healthcare professionals believed to have the power to authorise care preferences'**

*“I’d rather go as far the up ladder as I could. … Dr XYZ (GP) would have to go through various stages to get to that power. I mean I could talk to her (GP) very easily, she’s (GP) smashing. … But I think when it comes to this you want someone (clinician) who’s got [pause] both knowledgeable and can put into action what they are saying (respiratory consultant).”* (Patient 27 with severe COPD) (Tavares et al., 2020, p. 6)

##### 4.4.3.3 Feeling isolated

Particularly in institutional settings such as nursing homes, but also when people live at home, older people with chronic illness can feel a sense of social isolation described in one study as feeling ‘removed from life’.

**Code 'Feeling removed from life'**

Others feel that they are already, in significant respects, departed, irrevocably altered, or at a remove from life. *‘‘I wake at night, and say out loud, ‘I’m gone, I’m dead,’’’* said another patient and double amputee who, in previous conversations, had expressed feeling *‘‘stranded alive,’’* without family, without legs, with overwhelming debt, and without any sense of possibility for the future. (Russ et al., 2005, p. 315)

For some, isolation is rooted in previously mentioned losses (e.g. of physical mobility, tiredness, symptoms of illness) while for others, isolation results from feeling ignored or neglected by the people that surround them in daily life.

**Code 'Feeling ignored by healthcare professionals'**

Another patient (P5) described the situation as follows: *“I feel that I have become really isolated in this way of life … I lay like a package.”* These patients used metaphors like ‘furniture’ and ‘package’ and words of appraisal such as ‘isolated’ and ‘they aren’t that interested’. (Aasen et al., 2012, p. 65)

In the included studies, such feelings are particularly prevalent in nursing or care homes, but hospitals are also often considered isolating. Even older people who are not physically alone may experience profound loneliness. This experience is distinct from the loneliness experienced due to chronic illness at a younger age, as loneliness in older age is compounded by the loss of social networks caused by the deaths of loved ones. Conversely, when people are surrounded by loved ones, this helps them make sense of the hardships they are suffering due to ageing, chronic illness, and treatments. Some can also find comfort in spirituality and communication with deceased loved ones to make sense of their current situation and look towards the future with a sense of hope, or at least acceptance.

**Code 'Finding comfort in spirituality and communication with dead loved ones'**

In addition to conversation with God from her bed, May also engages in conversation with her husband through her grandfather’s clock, which chimes every quarter hour. During our interviews May would often stop talking to listen to the clock chime or even talk back to it, seeing it symbolically as a connection with her late husband, although quite aware he is dead. She once paused and said to the chimes, *“I love you, too, darling,”* explaining, *“My clock is talking to me …. He [my husband] spoils me rotten.”* (Vandenberg et al., 2018, p. 78)

#### 4.4.9 Behavioural dimension

##### 4.4.4.1 Minimising the impact of illness

Avoidance is a recurring strategy used to manage illness-related distress. Some participants minimise discomfort, delay seeking medical attention, or actively avoid discussions about their condition in order to retain a sense of normalcy. Especially people living with organ failure seem to favor a wait-and-see approach, conflicting with the expectations of healthcare professionals who often emphasise proactive decision-making and open dialogue about disease progression.

**Code '‘Wait and see’ approach'**

Nevertheless, patients and clinicians did not always agree on the urgency and attention that their CKD should be given over other priorities in patients' lives (quote 1f). Some patients expressed that they were best able to handle each issue if they addressed *“one thing at a time”*. Patients tended to take a *“wait and see”* approach with their CKD and relied on changes in clinical status to prompt them to refocus their efforts towards their CKD and preparation for dialysis or transplant. (House et al., 2022, p. 6)

Avoidance exists on a continuum, ranging from subtle acts of downplaying symptoms to outright denial of illness and impending death.

**Code 'Avoiding thoughts about death'**

*“I try not to think of that. I don’t know if it is that I don’t want to accept it or if I do it to survive.”* (Ek et al., 2011, p. 1483)

Acknowledgement of pain serves as an illustrative example – some people resist acknowledging pain, using other words such as ‘discomfort’. Older people can also assume a cheerful role or choose to utilise humour to deal with situations they feel are too difficult for themselves or others.

**Code 'Playing a cheerful role to manage'**

Some participants described having the social role of a cheerful patient at their dialysis clinic, a role which appeared to be helpful in managing their life with haemodialysis. *“I try to be a little positive. I joke with them and I talk about what they’ve done and what they’re up to at home and … and I enjoy that actually”.* On the other hand, this role could also be limiting. One participant was afraid that talking about worries with the nurses could be regarded as whining, and he thought he was supposed to be a happy patient. (Axelsson et al., 2012a, p. 48)

While this differs from outright avoidance, it can be a way to minimise the distressing consequences of situations such as stressful interactions with professional caregivers or emotional conversations with family and friends.

##### 4.4.4.2 Striving for normalcy in daily activities

Older people are continuously adapting to the consequences of illness and old age. In finding behavioural strategies to cope with the consequences of their illness and age, older people again strive for a continuous sense of self – in this context better termed as normalcy in daily activities. Strategies vary widely between people and across different points in a person's illness trajectory depending on personal factors, social context, and illness progression. Many older people maintain normalcy and continuity by engaging in familiar routines, preserving daily habits, and retaining meaningful activities such as gardening or looking after grandchildren. Engaging in behaviours that they consider ‘normal’ for themselves responds to the need for continuity between their past self and their current sense of self. Similarly, older people can redirect their attention to what remains within their control, whether through practical preparations (e.g. funeral planning) or focusing on short-term goals (e.g. reaching a wedding anniversary). Another way of retaining normalcy is to consciously accept help only when it is absolutely necessary. This last strategy is tied to the aforementioned striving for independence and control as a way to retain a continuous sense of self.

##### 4.4.4.3 Preserving hope

Regardless of the challenges faced by older people living with chronic illness, hope emerges as a persistent theme in participants' experiences. We placed ‘preserving hope’ in the behavioural dimension of the experience of chronic illness in old age because the participants in the included studies actively transform their hope into behaviours and attitudes such as focusing on the positive, on the present, on activities that are still possible, and on other people. They consciously take comfort in small joys, in the presence of loved ones, and in the belief that their legacy will persist beyond their lifetime.

**Code 'Funeral ceremony as acknowledgement of contributions in life'**

*“During the funeral ceremony, the contributions I have made to others and to society are acknowledged and made clear to the dependents in my families. I hope that this will inspire them to learn from my life. If my family participates in the ceremony and feels grateful to me, I will be able to earn more positive credits for my next life.”* (Shih et al., 2009, p. E35)

Hope is not necessarily tied to a cure or extended longevity (though this sometimes does remain important) but may manifest as hope for a ‘normal’ remaining life or a ‘good death’ – death that is painless, quick, and peaceful. Conversely, people imagine a ‘bad death’ to be painful, distressing, drawn-out, and dependent. While some older people consider shortening their lives or feel ready for death (see above), others still hope to live as long as possible. People hope for a good remaining life, and for a good death. In that sense, dying ‘in place’ is often cited as an ideal that older people hope for, though this preference is contingent on context; people living alone may prefer dying in a hospice or nursing or care home to an isolated death at home.

## 5 Discussion

Our synthesis highlights older people's active pursuit of continuity, familiarity, and normalcy to sustain a coherent sense of self in the context of chronic illness at the end of life. This finding shifts the focus from disruption caused by chronic or advanced illness to the dynamic interplay between loss and preservation of identity and meaning. The disruptive nature of chronic illness has long been recognised (Bury, 1982; Charmaz, 1983). Mike Bury conceptualised it as an experience that unsettles norms of reciprocity and mutual support within social networks, as well as plans for the future. He also found that chronic illness brings considerations of pain, suffering, and death into a person's consciousness, where these may have previously seemed remote (Bury, 1982, p. 169). Kathy Charmaz detailed the way in which chronic illness prompts a loss of self, rooted in “living a restricted life, existing in social isolation, experiencing discredited definitions of self and becoming a burden” (Charmaz, 1983, p. 169) – four themes that are echoed in this review's findings. This systematic review, however, shows a ubiquitous longing for continuity – across sense of self, social relationships, and daily activities – that both complements and extends existing theories conceptualising chronic illness as disruption (Llewellyn et al., 2014).

This review shows that older people's capacity to construct a continuous sense of self by creating meaningful and comforting connections depends on the physical, social, and contextual resources available to them, as well as on recognition by others. The importance of the environment in supporting personal capacity to deal with changes and hardships in old age has been emphasised previously (World Health Organization, 2020). In this context, others, such as professional and family caregivers, can provide “identity support” (Charmaz, 1994, p. 273) – a form of support that validates a person's sense of self. Our findings indeed show the crucial role family and friends play by offering emotional comfort and helping older people navigate complex healthcare systems. Yet, identity support extends beyond practical or emotional assistance. It emerges through interactions that affirm an older person's sense of continuity and uphold their identity across time. When family and friends engage with older people in ways that recognise and respect their continuous sense of self, they counteract ageist and ableist assumptions that life in old age with chronic illness is necessarily diminished, burdensome, or defined by decline. In healthcare settings, our findings show that when older people perceive their comorbidities and person-specific needs to be neglected by healthcare professionals, they feel reduced to “just an illness”, which has the opposite effect of identity support. Conversely, perceived empathy, attention, and recognition of their holistic self – including their past accomplishments, personal needs, and hopes for the future – brings emotional ease and represents a crucial form of identity support within care relationships.

Understanding experiences at the intersection of age and chronic illness offers insights into older people's distinct challenges and needs. For many older people there is no perceived separation between the ageing self and the chronically ill self as the experience of ageing is just as significant as the illness experience (Elofsson and Öhlén, 2004; Llewellyn et al., 2014). This interweaving shapes how chronic illness is understood and experienced in old age, and explains why some aspects of the chronic illness experience identified in this review appear specific to older people. For example, while the importance of being seen as a person beyond an illness has been described in younger people with chronic, life-threatening illnesses (Van Rickstal et al., 2022; Vandenbogaerde et al., 2024), the meanings of valued social roles differ in older age. Younger people may struggle with the loss of social roles such as their professional identity or that of family provider, whereas many older people have already transitioned out of such roles through life-stage changes. For the older people in this review, maintaining a sense of self and social roles therefore takes on meanings that resonate with later life, such as gardening, taking care of grandchildren, or leaving a legacy.

A second way in which the experience of chronic illness differs in older age concerns awareness of possible pain, suffering, and death. Such awareness, which is sometimes described as a disruptive consequence of chronic illness (Bury, 1982), seems to be less unexpected to older people. This might be because many, particularly the oldest old, associate ageing with illness and death, and have already contemplated the end of life (Hinck, 2007). However, our findings also show that the extent to which such awareness feels unexpected varies among people, influenced by factors such as the nature of the chronic illness. For example, while heart failure and frailty are seldom perceived as life-threatening by older people in this review, a diagnosis of incurable cancer or the perceived sudden transition from chronic kidney disease to kidney failure may mark a more abrupt confrontation with end-of-life awareness. Although public health authorities and healthcare providers widely frame cancer as a chronic illness (Firkins et al., 2020), for the older people in our review, this diagnosis still commonly provokes profound fear and despair, reflecting cancer's persistent association with abrupt end-of-life awareness and inevitable death (Kirby et al., 2020). In general, however, older people's responses to end-of-life awareness – whether expressed through denial, avoidance, acceptance, resistance, or negotiation – differ between people, and can shift over time.

Older people's responses to chronic illness, old age, and the awareness of a possible death in the (near) future, shift in relation to changing health, relationships, and personal priorities. Avoidance, for instance, can range from subtly downplaying symptoms to actively resisting discussions about illness or death, often as a means of retaining normalcy and emotional comfort. While this may help protect an older person's sense of self, it can also conflict with healthcare professionals' emphasis on proactive planning and open communication about disease progression. A recent thematic synthesis has highlighted the ‘total uncertainty’ of living with multimorbidity in older age, and identified people's active and passive responses to this uncertainty (Etkind et al., 2022). In our review, acceptance, resistance, and negotiation likewise reflect attempts to balance the realities of illness with the desire to preserve identity, autonomy, and valued roles. Importantly, these varied responses often coexist with hope, which emerges as a persistent feature of older people's accounts. The significant relation between hope and life with advanced chronic illness has also been documented in patient-caregiver dyads (Befecadu et al., 2024). In this sense, even strategies that appear avoidant can serve to protect and sustain hope, enabling older people to engage with life on their own terms. Viewed together, denial, avoidance, acceptance, resistance, negotiation, and hope can be understood not as fixed attitudes, but as fluid and adaptive strategies for managing the demands and uncertainty of living and dying with chronic illness in old age while preserving a continuous sense of self.

The same sense of fluidity should be applied to our understanding of older people's preferences with regards to their place of death. Beyond a desire to ‘die at home’, often referred to in existing literature (Pinto et al., 2024), this review shows a desire to ‘die in place’ with place representing much more than a physical location. Our results show that while many older people indicate they would prefer to die at home, what they seem to desire above all is to die in a place that feels familiar and safe. This is exemplified by people living alone at home who indicate they would rather die in hospice, and people in care or nursing homes who would rather remain there than die in hospital. Such preferences regarding place of death are a good example of end-of-life (care) preferences speaking on the one hand to people's need for continuity and familiarity, and on the other hand to the need to be surrounded and supported by others in difficult times. Regardless of the setting, the notion of a ‘good death’ is deeply personal and speaks to broader questions of people's sense of self – what it means to die with dignity, autonomy, and peace.

### 5.1 Implications

The findings of this review provide vital knowledge for guiding healthcare practice and policy that acknowledges not only the disruptions caused by illness but also the ways in which older people seek to preserve identity and meaning at the end of their lives. Our insights into the broad, multifaceted experience of chronic illness in old age also have relevance beyond healthcare practices and policies. They have implications for a better understanding of the society we live in, where dying in old age with chronic illness is becoming the norm (World Health Organization, 2024). We discuss the implications of our findings on personal, organisational, and societal levels before formulating recommendations for future research.

On the personal level, understanding older people's longing for continuity offers important guidance for family caregivers, healthcare professionals, and others involved in their care. Because older people are particularly at risk of social isolation (Fakoya et al., 2020), especially those living with chronic illness (Dahlberg et al., 2022), for many older people, family caregivers are not always available to provide consistent validation of their sense of self. This limits opportunities for identity support. In these circumstances, professional caregivers or volunteers may become their primary – sometimes their only – source of personal recognition and validation. This places healthcare professionals in a pivotal role: when they listen with empathy, acknowledge older people's past, and consider their health needs from a holistic perspective, they can help them sustain a continuous sense of self in the face of illness, ageing and dying. Conversely, when older people feel objectified or experience a loss of control in their interactions with healthcare professionals, the absence of family-based validation can render their sense of self especially vulnerable, making such experiences a source of profound emotional distress. Providing good end-of-life care not only calls for consideration for a person's chronic condition, their comorbidities and their non-medical concerns (Van den Block et al., 2025), but also requires time and consideration for their individuality, their temporality, and their social context.

On the organisational level, communities (e.g. neighbourhoods) and institutions (e.g. hospitals, care homes, social services) should recognise and support the ways in which older people seek to preserve identity and meaning. Research projects and interventions should focus on identifying optimal ways to build continuity and long-term relationships in the context of chronic illness in old age up until and beyond death (see Smets et al., 2024 for an example of current research). On the societal level, policy that supports older people who are living and dying with chronic illness will recognise and support their need for continuity in the personal, relational, and behavioural dimensions of experience. This requires moving beyond ageist and ableist discourses that frame old age and illness as disruption, decline, or burden, and instead recognising ageing as an inherent and continuous part of life, marked by enduring longing for meaning and connection. This can start with adapting the terminology used to describe older people and recognising that they are a highly heterogeneous group, contributing greatly to society and requiring attentive, empathic, and individual care and support (Makita et al., 2021).

### 5.2 Future research

While our review shows the richness of qualitative research on the experiences of older people nearing the end of life with chronic illness, we also identified substantial gaps in the existing literature. First, our findings highlight the centrality of temporality – how older people connect past, present, and future – in sustaining a coherent sense of self in the context of chronic illness, old age, and death. The longitudinal studies included in our review illustrate how the illness experience can evolve, for example showing how people's temporal awareness of past, present, and future chagnes as the end of life nears (Lindqvist et al., 2008). Prior work has also shown the importance of temporality and dynamics for understanding experiences of chronic illness (Joshi et al., 2025; Nicholson, 2009), but few of the studies included in this review used a longitudinal design, potentially missing important insights into how older people's experiences evolve over time.

Secondly, gender differences may play a role in how people negotiate dependence and associated emotions caused by old age and chronic illness (Charmaz, 1994). While the distribution of women (52%) and men (48%) among participants of the included studies in this review was almost equal, only one study provided an explicit reflection on gendered aspects of the chronic illness experience (Checa et al., 2020). We were therefore unable to expand on this topic, but it may be highly relevant for future research.

Thirdly, most studies included in this review were conducted in North-America or Europe, with a particular prevalence of studies conducted in Northern and Western Europe, therefore missing the experiences of a big part of the global population affected by chronic illness in old age. This pattern likely reflects both a broader shortage of research on this topic in other world regions (Pinto et al., 2024), as well as, to some extent, a limitation of our own inclusion criteria (see below).

Finally, our finding that most older people wish to ‘die in place’ but be surrounded and supported by others contributes to recent research suggesting that the ‘social space’ of death – constituted of social relations and meanings within the place of death – is at least as important to a person's well-being as the a geographical location of the place of death (Driessen et al., 2021). Future research should further explore the different meanings related to place of death, and to what extent the longing for continuity as described in this review can help us understand the significance of a place of death (see Namukwaya et al., 2024 for an example of current research).

### 5.3 Limitations

Our age-related inclusion criterion (limiting the sample to studies with all participants aged at least 65 or a mean age of at least 70) enabled us to focus on experiences specific to older people who have typically reached the age of retirement in most North-American and European countries. However, this criterion has potentially led to the exclusion of studies with mixed age-groups that might have offered relevant insights. Our inclusion criterion may also not reflect a typical age range for ‘older age’ in countries where life expectancy is lower (World Population Review, 2025), which could partly explain the limited number of studies from other parts of the world. We excluded books, book chapters, and monographs in this review for reasons detailed in Appendix A. This is a potential limitation of this review, as valuable qualitative research on chronic illness in old age is published in these formats (see Nicholson, 2009 for an example).

## 6 Conclusion

Despite the challenges that accompany ageing and chronic illness, older people who are nearing the end of life demonstrate remarkable resilience and agency in their efforts to sustain continuity in who they are. Rather than being defined solely by decline or disruption, their experience reveals ongoing capacities for adaptation, meaning-making, and connection. Their pursuit of continuity unfolds across personal, relational, and behavioural dimensions – through maintaining independence where possible, nurturing relationships, and preserving familiar routines and environments that anchor a sense of normalcy and purpose, including at the end of their lives.

Recognising these experiences calls for a comprehensive understanding of older people's lives that values not only their physical needs but also their emotional, social, and existential worlds. Supporting continuity of self in old age thus extends beyond healthcare: it involves creating social, cultural and institutional environments that affirm the worth, identity, and belonging of older people living with chronic illness. In this broader sense, promoting well-being at the end of life means fostering a society that recognises ageing not as withdrawal, but as an ongoing process of living meaningfully until the very end.

## Ethics approval

No ethics approval was required for this this systematic review of published studies.

## Funding

The authors disclose receipt of the following financial support for the research, authorship, and/or publication of this article: The TRAJECT project is funded by the 10.13039/501100000781European Research Council (ERC, Project No. 101077555). Views and opinions expressed are however those of the authors only and do not necessarily reflect those of the European Union or the European Research Council Executive Agency. Neither the European Union nor the granting authority can be held responsible for them. Emma Gobiet is the recipient of a PhD Fellowship fundamental research from the Research Foundation – Flanders (Grant No. 1157526N).

## CRediT authorship contribution statement

**Emma Gobiet:** Conceptualization, Data curation, Formal analysis, Methodology, Validation, Visualization, Writing – original draft. **Khyati Tripathi:** Conceptualization, Formal analysis, Methodology, Supervision, Validation, Writing – review & editing. **Aline De Vleminck:** Conceptualization, Methodology, Supervision, Validation, Writing – review & editing. **Lieve Van den Block:** Conceptualization, Methodology, Supervision, Validation, Writing – review & editing. **Lara Pivodic:** Conceptualization, Funding acquisition, Methodology, Supervision, Validation, Writing – review & editing.

## Declaration of competing interest

The authors declare no competing interests.

## Data Availability

No data was used for the research described in the article.

## Appendix A Justification of eligibility criteria

We included primary qualitative research or mixed-methods research in which qualitative data was separately reported describing the illness experiences of older people living with any chronic illness and nearing the end of life. This document provides in-depth descriptions and justifications of each of the concepts used in this eligibility statement.

1. **Study design:** Primary qualitative research or mixed methods-research in which qualitative research is reported separately.

We excluded systematic or scoping reviews, conference abstracts, news articles, models of care, letters, commentaries, study protocols, editorials, books, book chapters, monographs, non-peer-reviewed articles.

Most of these exclusions are usual for systematic reviews of primary research. We decided to exclude books, book chapters, and monographs because the methods of thematic synthesis require us to extract results from the ‘Results’ or ‘Findings’ sections of studies. This is very difficult to do in books, book chapters, and monographs hence we excluded them.

2. **Concepts studied:** Illness experiences.

The ‘experience’ criterion could only be a reason for exclusion if studies reported solely on the medical aspects of an illness experience (ex. pain management, physiological reaction to a medical procedure or intervention …).

We defined the sort of experiences that we included and came to following guidelines for screening:

We included studies reporting on experiences related to,

1. the illness itself,
2. healthcare,
3. social services,

Or,

4. emotional responses to the illness experience,
5. behavioural responses to the illness experience,
6. meaning-making processes: how older people make sense of their chronic illness experience(s) and give meaning to it.

We excluded studies reporting on,

- experiences specific to the COVID-19 pandemic
- physiological aspects of a condition, healthcare management systems or systems for disease diagnosis
- feasibility and acceptability studies, cost evaluations, intervention evaluations or evaluations of scales or assessment tools, discrete choice experiments and studies of a patient's choice for or against a treatment/procedure/intervention when they did not provide reasons for this choice.

3. **Population:** Older people.

Studies mentioning an age range of participants were included if the lower limit of the age range was ≥65 years old.

If no age range was given, we included studies mentioning an average or median age of ≥70 years old.

If no age range, median or mean was given, we included studies describing their population as ‘old(er)’ or ‘elderly’.

We excluded studies not fitting any of these criteria.

4. **Population:** Living with chronic illness.

We included all chronic illness diagnoses, including all types of dementia, frailty and multimorbidity.

The search string included general terms designating chronic disease as well as more specific terminology indicating the most common chronic diseases such as cancer, chronic obstructive pulmonary disease (COPD), chronic heart/kidney failure, neurodegenerative diseases or diabetes and asthma (World Health Organization, 2023). Frailty was also included in the study, as this can be considered a chronic illness and cause of death (Kojima et al., 2018).

5. **Population:** Nearing the end of life.

There is no universally accepted definition for the ‘end of life’. In this review, we consider the end of life to extend beyond the terminal phase of illness, therefore including studies with participants in advanced or severe stages of illness, and participants with inoperable or conservatively managed conditions. While they are not always described as nearing the end of life, their death is to be expected in the relatively near future, making their perspectives relevant to our understanding of experiences of chronic illness towards the end of life.

We included studies,

- With people nearing the end of life in the broadest sense (e.g. advanced, severe or end-stage of a specific disease or terminal illness, palliative or hospice care, end of life or any other description of the end of life given by the authors).
- Concerning advance care planning (ACP), conducted with older people living with chronic illness, even if the participants are not described as nearing the end of life; studies concerning ACP with healthy older adults are not included.
- Concerning the preferences and priorities concerning the end of life (of older people living with chronic illness) even if the participants are not described as nearing the end of life.
