# Supplementary material for: Longing for continuity: A systematic review and thematic synthesis of qualitative research on the experience of older people living with chronic illness towards the end of life
Source: Soc Sci Med. 2026 Jul;401:119220. doi: 10.1016/j.socscimed.2026.119220 (PMC13199950; doi:10.1016/j.socscimed.2026.119220)
Supplement: Multimedia component 1 [file mmc1.pdf]

Supplementary File 1: Search strings

PubMed

(“geriatr*”[tiab] OR “elderly”[tiab] OR “old people”[tiab] OR “old person*”[tiab] OR “Aging”[tiab] OR “Ageing”[tiab] OR “Aged”[tiab] OR “Aged 80, and over”[tiab] OR “Centenarian*”[tiab] OR “Nonagenerian*”[tiab] OR “Octogenerian*”[tiab] OR “older adult*”[tiab] OR “older people”[tiab] OR “aged person”[tiab] OR “older person”[tiab] OR “elderly person”[tiab] OR “elderly people”[tiab] OR “successful aging”[tiab] OR "Aged"[Mesh] OR "Aging"[Mesh]) AND

(“dement*”[tiab] OR “Dementia”[Mesh] OR “chronic illness*”[tiab] OR “chronic diseas*”[tiab] OR “noncommunicable diseas*”[tiab] OR “non-communicable diseas*”[tiab] OR “non-infectious diseas*”[tiab] OR “noninfectious illness*”[tiab] OR “noncommunicable illness*”[tiab] OR “non-communicable illness*”[tiab] OR “non-infectious illness*”[tiab] OR “noninfectious diseas*”[tiab] OR "Chronic Disease"[Mesh] OR "Noncommunicable Diseases"[Mesh] OR “cancer*”[tiab] OR "Neoplasms"[Mesh] OR “neoplasm*”[tiab] OR “cardiovascular diseas*”[tiab] OR "Cardiovascular Diseases"[Mesh] OR “chronic heart failure”[tiab] OR "Pulmonary Disease, Chronic Obstructive"[Mesh] OR “chronic obstructive lung diseas*”[tiab] OR “chronic obstructive pulmonary diseas*”[tiab] OR "Diabetes Mellitus"[Mesh] OR “diabetes”[tiab] OR "Asthma"[Mesh] OR “asthma”[tiab] OR “chronic kidney failure”[tiab] OR “neurodegenerative diseas*”[tiab] OR “chronic renal insufficienc*”[tiab] OR "Renal Insufficiency, Chronic"[Mesh] OR "Kidney Failure, Chronic"[Mesh] OR "Neurodegenerative Diseases"[Mesh]) AND

(“qualitative research”[tiab] OR “qualitative method*”[tiab] OR “interview*”[tiab] OR “ethnograph*”[tiab] OR “focus group*”[tiab] OR “grounded theor*”[tiab] OR “mixed method*”[tiab] OR “case stud*”[tiab] OR “participant observation*”[tiab] OR “observant participation*”[tiab] OR “content analys*”[tiab] OR “thematic analys*”[tiab] OR “phenomenolog*”[tiab] OR "Qualitative Research"[Mesh] OR "Grounded Theory"[Mesh] OR "Focus Groups"[Mesh]) AND

(“patient perspective*”[tiab] OR “quality-of-life”[tiab] OR "Patient Preference*"[tiab] OR "Quality of Life"[tiab] OR “Life Quality”[tiab] OR “HRQOL”[tiab] OR “QOL”[tiab] OR “care need*”[tiab] OR “well-being”[tiab] OR “wellbeing”[tiab] OR "Quality of Life"[Mesh] OR “lived experience*”[tiab] OR "Patient Preference"[Mesh]) AND

(“palliative care”[tiab] OR “Palliative Care”[Mesh] OR “palliative”[tiab] OR “terminal phase*”[tiab] OR “terminal illness*”[tiab] OR "Terminally Ill"[Mesh] OR “terminally ill”[tiab] OR “terminal care”[tiab] OR “Terminal Care”[Mesh] OR “terminal diseas*”[tiab] OR “death”[tiab] OR “Death”[Mesh:NoExp] OR “end-of-life care”[tiab] OR “end-of-life”[tiab] OR “end of life”[tiab] OR “advance care planning”[tiab] OR “ACP”[tiab] OR “EOL”[tiab] OR "Advance Care Planning"[Mesh] OR “frail*”[tiab] OR “advanced heart failure”[tiab] OR “Frailty”[Mesh] OR “end stage renal failure”[tiab] OR “end stage kidney disease*”[tiab] OR “end stage renal disease*”[tiab] OR “end stage chronic obstructive pulmonary disease*”[tiab] OR “advanced cancer*”[tiab])

Embase

(‘aged’/exp OR ‘aging’/exp OR ‘geriatr*’:ti,ab,kw OR ‘elderly’:ti,ab,kw OR ‘old people’:ti,ab,kw OR ‘old person*’:ti,ab,kw OR ‘Aging’:ti,ab,kw OR ‘Ageing’:ti,ab,kw OR ‘Aged’:ti,ab,kw OR ‘Aged 80, and over’:ti,ab,kw OR ‘Centenarian*’:ti,ab,kw OR ‘Nonagenerian*’:ti,ab,kw OR ‘Octogenerian*’:ti,ab,kw OR ‘older adult*’:ti,ab,kw OR ‘older people’:ti,ab,kw OR ‘aged person’:ti,ab,kw OR ‘older person’:ti,ab,kw OR ‘elderly person’:ti,ab,kw OR ‘elderly people’:ti,ab,kw OR ‘successful aging’:ti,ab,kw) AND

(‘degenerative disease’/exp OR ‘chronic kidney failure’/exp OR ‘asthma’ OR ‘diabetes mellitus’ OR ‘chronic obstructive lung disease’ OR ‘cardiovascular disease’ OR ‘vascular disease’ OR ‘heart disease’ OR ‘neoplasm’ OR ‘non communicable disease’ OR ‘chronic disease’ OR ‘multiple chronic conditions’ OR ‘dementia’/exp OR ‘dement*’:ti,ab,kw OR ‘chronic illness*’:ti,ab,kw OR ‘chronic diseas*’:ti,ab,kw OR ‘noncommunicable diseas*’:ti,ab,kw OR ‘non-communicable diseas*’:ti,ab,kw OR ‘non-infectious diseas*’:ti,ab,kw OR ‘noninfectious diseas*’:ti,ab,kw OR ‘noncommunicable illness*’:ti,ab,kw OR ‘non-communicable illness*’:ti,ab,kw OR ‘non-infectious illness*’:ti,ab,kw OR ‘noninfectious illness*’:ti,ab,kw OR ‘cancer*’:ti,ab,kw OR ‘neoplasm*’:ti,ab,kw OR ‘cardiovascular diseas*’:ti,ab,kw OR ‘chronic obstructive lung diseas*’:ti,ab,kw OR ‘chronic obstructive pulmonary diseas*’:ti,ab,kw OR ‘diabetes’:ti,ab,kw OR ‘asthma’:ti,ab,kw OR ‘chronic kidney failure’:ti,ab,kw OR ‘neurodegenerative diseas*’:ti,ab,kw OR ‘chronic heart failure’:ti,ab,kw) AND

(‘grounded theory’ OR ‘qualitative research’/exp OR ‘qualitative research’:ti,ab,kw OR ‘qualitative method*’:ti,ab,kw OR ‘interview*’:ti,ab,kw OR ‘ethnograph*’:ti,ab,kw OR ‘focus group*’:ti,ab,kw OR ‘grounded theor*’:ti,ab,kw OR ‘mixed method*’:ti,ab,kw OR ‘case stud*’:ti,ab,kw OR ‘participant observation*’:ti,ab,kw OR ‘observant participation*’:ti,ab,kw OR ‘content analys*’:ti,ab,kw OR ‘thematic analys*’:ti,ab,kw OR ‘phenomenolog*’:ti,ab,kw) AND

(‘patient preference’ OR ‘quality of life’ OR ‘patient perspective*’:ti,ab,kw OR ‘quality-of-life’:ti,ab,kw OR ‘Patient Preference*’:ti,ab,kw OR ‘Quality of Life’:ti,ab,kw OR ‘Life Quality’:ti,ab,kw OR ‘HRQOL’:ti,ab,kw OR ‘QOL’:ti,ab,kw OR ‘care need*’:ti,ab,kw OR ‘well-being’:ti,ab,kw OR ‘wellbeing’:ti,ab,kw OR ‘lived experience*’:ti,ab,kw) AND

(‘frailty’ OR ‘advance care planning’ OR ‘death’ OR ‘terminal care’/exp OR ‘terminally ill patient’/exp OR ‘palliative therapy’/exp OR ‘palliative nursing’ OR ‘palliative care’:ti,ab,kw OR ‘palliative’:ti,ab,kw OR ‘terminal phase*’:ti,ab,kw OR ‘terminal illness*’:ti,ab,kw OR ‘terminally ill’:ti,ab,kw OR ‘terminal care’:ti,ab,kw OR ‘terminal diseas*’:ti,ab,kw OR ‘death’:ti,ab,kw OR ‘end-of-life care’:ti,ab,kw OR ‘end-of-life’:ti,ab,kw OR ‘end of life’:ti,ab,kw OR ‘advance care planning’:ti,ab,kw OR ‘ACP’:ti,ab,kw OR ‘EOL’:ti,ab,kw OR ‘advanced heart failure’:ti,ab,kw OR ‘frail*’:ti,ab,kw OR ‘end stage renal failure’:ti,ab,kw OR ‘end stage kidney disease*’:ti,ab,kw OR ‘end stage renal disease*’:ti,ab,kw OR ‘end stage chronic obstructive pulmonary disease*’:ti,ab,kw OR ‘advanced cancer’:ti,ab,kw)

PsycInfo

(TI,AB,SU(“geriatr*”) OR TI,AB,SU(“elderly”) OR TI,AB,SU(“old people”) OR TI,AB,SU(“old person*”) OR TI,AB,SU(“Aging”[tiab]) OR TI,AB,SU(“Ageing”) OR TI,AB,SU(“Aged”) OR TI,AB,SU(“Aged 80, and over”) OR TI,AB,SU(“Centenarian*”) OR TI,AB,SU(“Nonagenerian*”) OR TI,AB,SU(“Octogenerian*”) OR TI,AB,SU(“older adult*”) OR TI,AB,SU(“older people”) OR TI,AB,SU(“aged person”) OR TI,AB,SU(“older person”) OR TI,AB,SU(“elderly person”) OR TI,AB,SU(“elderly people”) OR TI,AB,SU(“successful aging”))

AND

(TI,AB,SU(“dement*”) OR TI,AB,SU(“chronic illness*”) OR TI,AB,SU(“chronic diseas*”) OR TI,AB,SU(“noncommunicable diseas*”) OR TI,AB,SU(“non-communicable diseas*”) OR TI,AB,SU(“non-infectious diseas*”) OR TI,AB,SU(“noninfectious diseas*”) OR TI,AB,SU(“noncommunicable illness*”) OR TI,AB,SU(“non-communicable illness*”) OR TI,AB,SU(“non-infectious illness*”) OR TI,AB,SU(“noninfectious illness*”) OR TI,AB,SU(“cancer*”) OR TI,AB,SU(“neoplasm*”) OR TI,AB,SU(“cardiovascular diseas*”) OR TI,AB,SU(“chronic obstructive lung diseas*”) OR TI,AB,SU(“chronic obstructive pulmonary diseas*”) OR TI,AB,SU(“diabetes”) OR TI,AB,SU(“asthma”) OR TI,AB,SU(“chronic kidney failure”) OR TI,AB,SU(“neurodegenerative diseas*”) OR TI,AB,SU(“chronic renal insufficienc*”) OR TI,AB,SU(“chronic heart failure”))

AND

(TI,AB,SU(“qualitative research”) OR TI,AB,SU(“qualitative method*”) OR TI,AB,SU(“interview*”) OR TI,AB,SU(“ethnograph*”) OR TI,AB,SU(“focus group*”) OR TI,AB,SU(“grounded theor*”) OR TI,AB,SU(“mixed method*”) OR TI,AB,SU(“case stud*”) OR TI,AB,SU(“participant observation*”) OR TI,AB,SU(“observant participation*”) OR TI,AB,SU(“content analys*”) OR TI,AB,SU(“thematic analys*”) OR TI,AB,SU(“phenomenolog*”))

AND

(TI,AB,SU(“patient perspective*”) OR TI,AB,SU(“quality-of-life”) OR TI,AB,SU("Patient Preference*") OR TI,AB,SU("Quality of Life") OR TI,AB,SU(“Life Quality”) OR TI,AB,SU(“HRQOL”) OR TI,AB,SU(“QOL”) OR TI,AB,SU(“care need*”) OR TI,AB,SU(“well-being”) OR TI,AB,SU(“wellbeing”) OR TI,AB,SU(“lived experience*”))

AND

(TI,AB,SU(“palliative care”) OR TI,AB,SU(“palliative”) OR TI,AB,SU(“terminal phase*”) OR TI,AB,SU(“terminal illness*”) OR TI,AB,SU(“terminally ill”) OR TI,AB,SU(“terminal care”) OR TI,AB,SU(“terminal diseas*”) OR TI,AB,SU(“death”) OR TI,AB,SU(“end-of-life care”) OR TI,AB,SU(“end-of-life”) OR TI,AB,SU(“end of life”) OR TI,AB,SU(“advance care planning”) OR TI,AB,SU(“ACP”) OR TI,AB,SU(“EOL”) OR TI,AB,SU(“frail*”) OR TI,AB,SU(“advanced heart failure”) OR TI,AB,SU(“end stage renal failure”) OR TI,AB,SU(“end stage kidney disease*”) OR TI,AB,SU(“end stage renal disease*”) OR TI,AB,SU(“end stage chronic obstructive pulmonary disease*”) OR TI,AB,SU(“advanced cancer*”))

Web Of Science (Core Collection)

(“geriatr*” OR “elderly” OR “old people” OR “old person*” OR “Aging” OR “Ageing” OR “Aged” OR “Aged 80, and over” OR “Centenarian*” OR “Nonagenerian*” OR “Octogenerian*” OR “older adult*” OR “older people” OR “aged person” OR “older person” OR “elderly person” OR “elderly people” OR “successful aging”)AND

(“dement*” OR “chronic illness*” OR “chronic diseas*” OR “noncommunicable diseas*” OR “non-communicable diseas*” OR “non-infectious diseas*” OR “noninfectious diseas*” OR “noncommunicable illness*” OR “non-communicable illness*” OR “non-infectious illness*” OR “noninfectious illness*” OR “cancer*” OR “neoplasm*” OR “cardiovascular diseas*” OR “chronic heart failure” OR “chronic obstructive lung diseas*” OR “chronic obstructive pulmonary diseas*” OR “diabetes” OR “asthma” OR “chronic kidney failure” OR “neurodegenerative diseas*” OR “chronic renal insufficienc*”) AND

(“qualitative research” OR “qualitative method*” OR “interview*” OR “ethnograph*” OR “focus group*” OR “grounded theor*” OR “mixed method*” OR “case stud*” OR “participant observation*” OR “observant participation*” OR “content analys*” OR “thematic analys*” OR “phenomenolog*”) AND

(“patient perspective*” OR “quality-of-life” OR "Patient Preference*" OR "Quality of Life" OR “Life Quality” OR “HRQOL” OR “QOL” OR “care need*” OR “well-being” OR “wellbeing” OR “lived experience*”) AND

(“palliative care” OR “palliative” OR “terminal phase*” OR “terminal illness*” OR “terminally ill” OR “terminal care” OR “terminal diseas*” OR “death” OR “end-of-life care” OR “end-of-life” OR “end of life” OR “advance care planning” OR “ACP” OR “EOL” OR “frail*” OR “advanced heart failure” OR “end stage renal failure” OR “end stage kidney disease*” OR “end stage renal disease*” OR “end stage chronic obstructive pulmonary disease*” OR “advanced cancer*”)
