# Supplementary material for: Longing for continuity: A systematic review and thematic synthesis of qualitative research on the experience of older people living with chronic illness towards the end of life
Source: Soc Sci Med. 2026 Jul;401:119220. doi: 10.1016/j.socscimed.2026.119220 (PMC13199950; doi:10.1016/j.socscimed.2026.119220)
Supplement: Multimedia component 2 [file mmc2.pdf]

Supplementary File 2: Quality appraisal

CASP Item		Yes	No	Can't tell
1	Was there a clear statement of the research aim?	[1-29]		[30, 31]
2	Is a qualitative methodology appropriate?	[1-31]		
3	Was the research design appropriate to address the aims of the research?	[1-4, 6-10, 12, 15, 18-25, 27-30]	[5]	[11, 13, 14, 16, 17, 26, 31]
4	Was the recruitment strategy appropriate to the aims of the research?	[1-8, 10, 11, 13-16, 18, 20-25, 28, 30]		[9, 12, 17, 19, 26, 27, 29, 31]
5	Was the data collected in a way that addressed the research issue?	[1-31]		
6	Has the relationship between researcher and participants been adequately considered?	[1, 20, 22, 23]	[5, 6, 9, 10, 13, 16, 19, 25-27, 30, 31]	[2-4, 7, 8, 11, 12, 14, 15, 17, 18, 21, 24, 28, 29]
7	Have ethical issues been taken into consideration?	[1-11, 14-16, 18, 20-24, 26, 28-30]	[19]	[12, 13, 17, 25, 27, 31]
8	Was the data analysis sufficiently rigorous?	[1-5, 7-13, 15-22, 24-28, 30]		[6, 14, 23, 29, 31]
9	Is there a clear statement of findings?	[1-28, 30]		[29, 31]
10	How valuable is the research?			

1. Aasen, E.M., M. Kvangarsnes, and K. Heggen, *Perceptions of patient participation amongst elderly patients with end-stage renal disease in a dialysis unit.* Scand J Caring Sci, 2012. **26**(1): p. 61-9.
2. Axelsson, L., et al., *Living with haemodialysis when nearing end of life.* Scand J Caring Sci, 2012. **26**(1): p. 45-52.
3. Axelsson, L., et al., *Thoughts on death and dying when living with haemodialysis approaching end of life.* JOURNAL OF CLINICAL NURSING, 2012. **21**(15-16): p. 2149-2159.
4. Bristowe, K., et al., *Invisible and intangible illness: a qualitative interview study of patients' experiences and understandings of conservatively managed end-stage kidney disease.* Ann Palliat Med, 2019. **8**(2): p. 121-129.
5. Checa, C., et al., *Living with advanced heart failure: A qualitative study.* PLoS One, 2020. **15**(12): p. e0243974.
6. Cortis, J.D. and A. Williams, *Palliative and supportive needs of older adults with heart failure.* Int Nurs Rev, 2007. **54**(3): p. 263-70.
7. Dale, M.J. and B. Johnston, *An exploration of the concerns of patients with inoperable lung cancer.* Int J Palliat Nurs, 2011. **17**(6): p. 285-90.
8. Devik, S.A., et al., *Meanings of being old, living on one's own and suffering from incurable cancer in rural Norway.* European Journal of Oncology Nursing, 2013. **17**(6): p. 781-787.
9. Dunham, M., P. Allmark, and K. Collins, *Older people's experiences of cancer pain: a qualitative study.* Nurs Older People, 2017. **29**(6): p. 28-32.

10. Ek, K., et al., *Struggling to retain living space: Patients' stories about living with advanced chronic obstructive pulmonary disease*. Journal of Advanced Nursing, 2011. **67**(7): p. 1480-1490.
11. Franklin, L.L., B.M. Ternestedt, and L. Nordenfelt, *Views on dignity of elderly nursing home residents*. Nursing Ethics, 2006. **13**(2): p. 130-146.
12. Frazer, M.S. and P. Mobley, *A mixed methods analysis of quality of life among late-life patients diagnosed with chronic illnesses.* Health Qual Life Outcomes, 2017. **15**(1): p. 222.
13. Gerlich, M.G., et al., *'Who is going to explain it to me so that I understand?' Health care needs and experiences of older patients with advanced heart failure.* European Journal of Ageing, 2012. **9**(4): p. 297-303.
14. Goodman, C., et al., *Preferences and priorities for ongoing and end-of-life care: A qualitative study of older people with dementia resident in care homes*. International Journal of Nursing Studies, 2013. **50**(12): p. 1639-1647.
15. Holmberg, B. and T. Godskesen, *Dignity in bodily care at the end of life in a nursing home: an ethnographic study.* Bmc Geriatrics, 2022. **22**(1).
16. House, T.R., et al., *Challenges to Shared Decision Making About Treatment of Advanced CKD: A Qualitative Study of Patients and Clinicians*. American Journal of Kidney Diseases, 2022. **79**(5): p. 657-666.e1.
17. Jespersen, E., L.R. Minet, and N. Nissen, *Symptoms of total pain experienced by older people with advanced gastrointestinal cancer receiving palliative chemotherapy.* Eur J Cancer Care (Engl), 2022. **31**(6): p. e13674.
18. Klindtworth, K., et al., *Living with and dying from advanced heart failure: understanding the needs of older patients at the end of life*. BMC Geriatr, 2015. **15**: p. 125.
19. Lindhardt, C.L., et al., *Information provision to older patients receiving palliative chemotherapy: A quality study.* BMJ Supportive and Palliative Care, 2021.
20. Lloyd, A., et al., *Physical, social, psychological and existential trajectories of loss and adaptation towards the end of life for older people living with frailty: A serial interview study.* BMC Geriatrics, 2016. **16**(1): p. 176.
21. Selman, L., et al., *The views and experiences of older people with conservatively managed renal failure: A qualitative study of communication, information and decision-making.* Palliative Medicine, 2018. **32**(1): p. 244.
22. Shih, F.J., et al., *Spiritual needs of Taiwan's older patients with terminal cancer.* Oncol Nurs Forum, 2009. **36**(1): p. E31-8.
23. Tavares, N., et al., *The preferences of patients with chronic obstructive pulmonary disease are to discuss palliative care plans with familiar respiratory clinicians, but to delay conversations until their condition deteriorates: A study guided by interpretative phenomenological analysis.* Palliative Medicine, 2020. **34**(10): p. 1361-1373.
24. van Gurp, J.L.P., et al., *Living and dying with incurable cancer: a qualitative study on older patients' life values and healthcare professionals' responsivity.* BMC Palliat Care, 2020. **19**(1): p. 109.
25. Vandenberg, A.E., et al., *Contours of "here": Phenomenology of space for assisted living residents approaching end of life.* Journal of Aging Studies, 2018. **47**: p. 72-83.
26. Warrén Stomberg, M., *Guest at hospice: time for consideration.* Am J Hosp Palliat Care, 2009. **26**(4): p. 277-80.
27. Murali, K.P., et al., *"There Should Be A Nurse On Call": Complex Care Needs of Low-Income Older Adults in Medicaid-Supported Assisted Living*. Journal of Palliative Medicine, 2025. **28**(8): p. 1029-1037.
28. Hole, B., et al., *'It's basically 'have that or die'': a qualitative study of older patients' choices between dialysis and conservative kidney management*. BMJ Open, 2025. **15**(3): p. e095185.

29. Sugiyama, R. and Y. Nakamura, *The Lives of Older People With Advanced Cancer Who Live Alone During Outpatient Cancer Chemotherapy: A Descriptive Qualitative Study.* Nurs Health Sci, 2025. **27**(1): p. e70076.
30. Lindqvist, O., et al., *Time and bodily changes in advanced prostate cancer: talk about time as death approaches.* J Pain Symptom Manage, 2008. **36**(6): p. 648-56.
31. Russ, A.J., J.K. Shim, and S.R. Kaufman, *"Is there life on dialysis?": time and aging in a clinically sustained existence.* Med Anthropol, 2005. **24**(4): p. 297-324.
