# Supplementary material for: Longing for continuity: A systematic review and thematic synthesis of qualitative research on the experience of older people living with chronic illness towards the end of life
Source: Soc Sci Med. 2026 Jul;401:119220. doi: 10.1016/j.socscimed.2026.119220 (PMC13199950; doi:10.1016/j.socscimed.2026.119220)
Supplement: Multimedia component 3 [file mmc3.pdf]

Supplementary File 3: Mind map

The mind map starts from a central idea and recurring notion we identified in this review that many older people with serious chronic illness report a general sense of loss which is rooted in bodily experiences caused by chronic illness and age. The mind map subsequently expands and branches out into six main sections that illustrate associated ideas:

1) the kinds of losses people experience caused by illness and age (e.g. loss of control, mobility, future plans, time),
2) how they experience the dependence imposed on them by illness and age (e.g. feeling guilty, embarrassed, like a burden),
3) how losses are tied to experiences with and expectations from healthcare systems and healthcare professionals (e.g. feeling grateful when there is empathy and continuity in care),
4) the role of family and friends in older people's experiences of illness (e.g. offering crucial support and giving purpose to life regardless illness),
5) how people look at their future with illness (e.g. as worrying, or as associated with death),
6) the coping and managing strategies people use to deal with the losses they experience due to illness and age (e.g. managing with humor, finding comfort in spirituality, focusing on the present, and holding on to hope).

Anyone can access the mind map through this link:
https://app.xmind.com/share/f9ARi72z?xid=ZDLQXPle

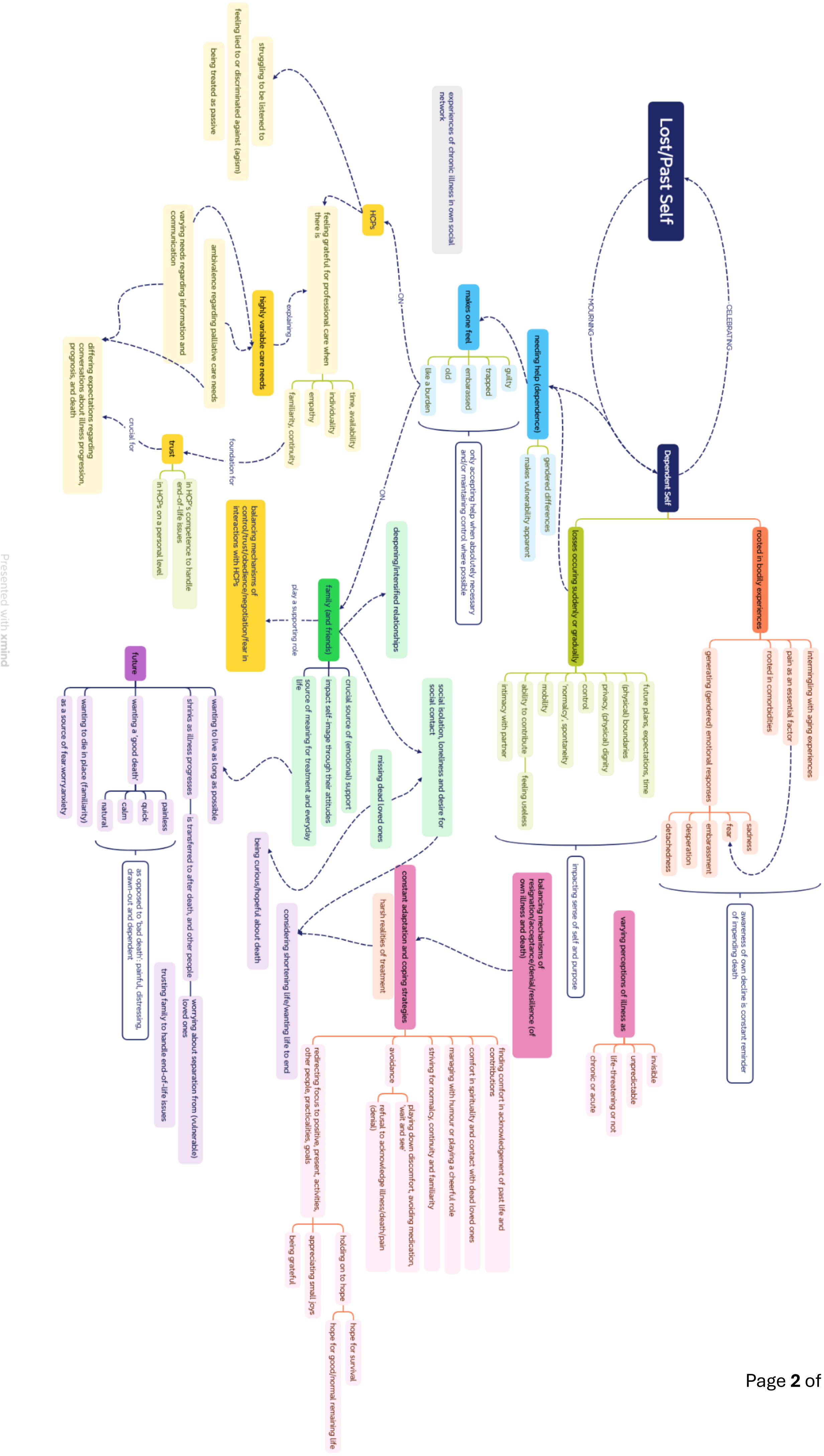
Lost/Past Self
CELEBRATING
MOURNING
Dependent Self
rooted in bodily experiences
intermingling with aging experiences
pain as an essential factor
rooted in comorbidities
generating (gendered) emotional responses
sadness
fear
embarassment
desperation
detachedness
awareness of own decline is constant reminder of impending death
losses occuring suddenly or gradually
future plans, expectations, time
(physical) boundaries
privacy, (physical) dignity
control
'normalcy', spontaneity
mobility
ability to contribute
feeling useless
intimacy with partner
impacting sense of self and purpose
needing help (dependence)
gendered differences
makes vulnerability apparent
makes one feel
guilty
trapped
embarassed
old
like a burden
only accepting help when absolutely necessary and/or maintaining control where possible
experiences of chronic illness in own social network
HCPs
ON
struggling to be listened to
feeling lied to or discriminated against (agism)
being treated as passive
feeling grateful for professional care when there is
time, availability
individuality
empathy
familiarity, continuity
explaining
highly variable care needs
ambivalence regarding palliative care needs
varying needs regarding information and communication
differing expectations regarding conversations about illness progression, prognosis, and death
trust
crucial for
foundation for
in HCP's competence to handle end-of-life issues
in HCPs on a personal level
ON
family (and friends)
play a supporting role
balancing mechanisms of control/trust/obedience/negotiation/fear in interactions with HCPs
deepening/intensified relationships
crucial source of (emotional) support
impact self-image through their attitudes
source of meaning for treatment and everyday life
social isolation, loneliness and desire for social contact
missing dead loved ones
being curious/hopeful about death
considering shortening life/wanting life to end
constant adaptation and coping strategies
harsh realities of treatment
balancing mechanisms of resignation/acceptance/denial/resilience (of own illness and death)
varying perceptions of illness as
invisible
unpredictable
life-threatening or not
chronic or acute
finding comfort in acknowledgement of past life and contributions
comfort in spirituality and contact with dead loved ones
managing with humour or playing a cheerful role
striving for normalcy, continuity and familiarity
avoidance
playing down discomfort, avoiding medication, 'wait and see'
refusal to acknowledge illness/death/pain (denial)
redirecting focus to positive, present, activities, other people, practicalities, goals
holding on to hope
hope for survival
hope for good/normal remaining life
appreciating small joys
being grateful
future
wanting to live as long as possible
shrinks as illness progresses
is transferred to after death, and other people
worrying about separation from (vulnerable) loved ones
wanting a 'good death'
painless
quick
calm
natural
as opposed to 'bad death': painful, distressing, drawn-out and dependent
wanting to die in place (familiarity)
as a source of fear/worry/anxiety
trusting family to handle end-of-life issues
Presented with xmind
