# Supplementary material for: Longing for continuity: A systematic review and thematic synthesis of qualitative research on the experience of older people living with chronic illness towards the end of life
Source: Soc Sci Med. 2026 Jul;401:119220. doi: 10.1016/j.socscimed.2026.119220 (PMC13199950; doi:10.1016/j.socscimed.2026.119220)
Supplement: Multimedia component 4 [file mmc4.docx]

Supplementary File 4: Generating analytical themes

In step 3 of our thematic synthesis, we inferred overarching, analytical themes starting from the mind map in step 2 (see Supplementary File 3). As an example, we describe here how we came to the overarching theme ‘Longing for independence while relying on others’ and how it fit into the relational dimension of the experience of chronic illness.

We describe three aspects of this interpretative process of analysis. They should not be seen as a linear succession of steps to be followed chronologically, but as an iterative process during which the researchers move between aspects until the results as presented in the manuscripts are finalised.

1. The main aspect of step 3 was seeing and creating stories within the mind map. One of the stories we deemed important started from the top of the mind map – in the bodily experiences and the losses experienced – and then flowed to what those experiences mean for relationships with others in the lower left part of the mind map. There, needing help (and thus experiencing dependence) influenced the participants’ relationships with and expectations of the people surrounding them (i.e. healthcare professionals, family and friends). After creating a coherent story from the mind map, we tried to establish what the core pattern of meaning in that story was to us. In this example, ‘Longing for independence while relying on others’ encapsulated this meaning, and became the overarching, analytical theme.


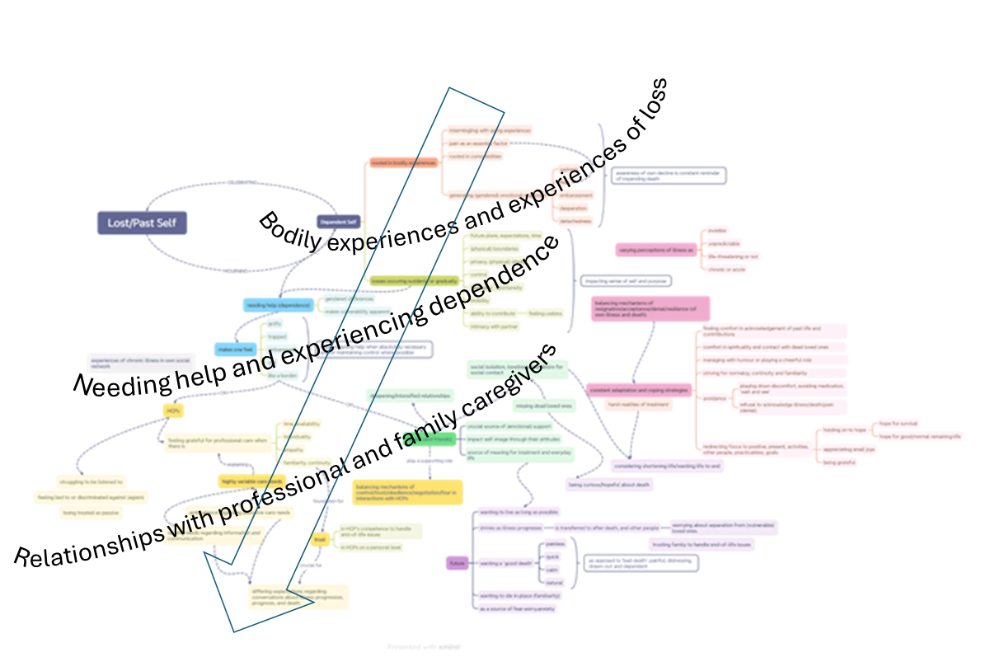
The following image shows how this storyline is situated within the mind map:

1. We made sure that the entirety of the mind map was covered by our overarching themes. This is how we ended up with 6 overarching themes, organised over the personal, relational and behavioural dimensions of the experience of chronic illness.
2. We linked our stories and overarching themes back to the original codes. We thus went back to the 385 codes and made sure that each of them made sense within our results, i.e. that each of them fit into at least one overarching theme.
